# Changes in Caries Risk and Activity of a 9-Year-Old Patient with Niemann-Pick Disease Type C

**DOI:** 10.1155/2015/571098

**Published:** 2015-01-01

**Authors:** Késsia Suênia Fidelis Mesquita-Guimarães, Andiara De Rossi, Aldevina Campos Freitas, Paulo Nelson-Filho, Raquel Assed da Silva, Alexandra Mussolino de Queiroz

**Affiliations:** Department of Pediatric Clinics Dentistry, Faculty of Dentistry of Ribeirão Preto, University of São Paulo, Avenida do Café, s/n, Monte Alegre, 14040-904 Ribeirão Preto, SP, Brazil

## Abstract

*Objective.* This case report describes the changes in caries risk and activity and dental treatment of a 9-year-old patient who presented with signs and symptoms of Niemann-Pick disease type C (NPC). *Treatment.* The preventive dental treatment included instructions to caregivers for oral hygiene and diet. A calcium hydroxide pulpotomy and restorative dental treatments were performed in a dental office with desensitization techniques and behavioral management. The patient was attended every 3 months for the control of dental plaque biofilm, for topical fluoride application, and for observing the pulpotomized tooth. *Results.* The bacterial plaque biofilm was being adequately controlled by the caregiver. After 2 years, the clinical and radiographic examination of the pulpotomized tooth showed the absence of internal root resorption and bone rarefaction, and clinical examination showed tooth sensitivity, dental pain, and gingival swelling. *Conclusion.* The pulpotomy prevented clinical and radiographic success. Dentists must be aware of and be able to identify systemic and local aspects associated with caries risk of children with NPC disease. Furthermore, dentists must employ stringent preventive measures and provide instructions to caregivers to reduce caries risk.

## 1. Introduction

The Niemann-Pick disease types A and B are caused by acid sphingomyelinase deficiency that results from mutations in the SMPD1 gene. Niemann-Pick disease type C (NPC) and type D result from mutations in either the NPC1 or the NPC2 gene [1], and these types erroneously induce cholesterol transport [2]. NPC is characterized by the accumulation of cholesterol and sphingolipids in neuronal endosomes and lysosomes [2]. Mutations in the NPC1 gene occur in 95% of cases [3, 4]. Niemann-Pick disease is fatal [5], hereditary, and autosomal recessive [2, 6, 7]. The disease occurs in 1/120,000 live births [4].

The initial clinic manifestation of NPC may occur in the late-infantile and juvenile period (approximately 60–70% of cases). The manifestations include hepatic, neurologic, or psychiatric signals and symptoms [3]. The most common neurological characteristic of the disease is vertical supranuclear gaze palsy [4] accompanied by progressive cognitive deterioration [8]. Changes in the liver, including common neonatal icterus [3, 4] and hepatosplenomegaly [3, 4, 9], are often present during the first months of life.

Caries disease is multifactorial and involves the host, microorganisms, and diet [10]; thus, any change in these factors can alter the individual caries risk and activity. Systemic conditions can also alter caries risk and activity [11].

Dentists can contribute to the early diagnosis of a variety of diseases and develop preventive and restorative dental care strategies for children with special health care needs. Early diagnosis results in improved quality of life for the patient. Therefore, the aim of this case report was to describe the changes in caries etiology in a 9-year-old patient who developed signs and symptoms of NPC disease. This report also describes the methods that were adopted during the dental treatment.

## 2. Case Report

**Figure 1:**
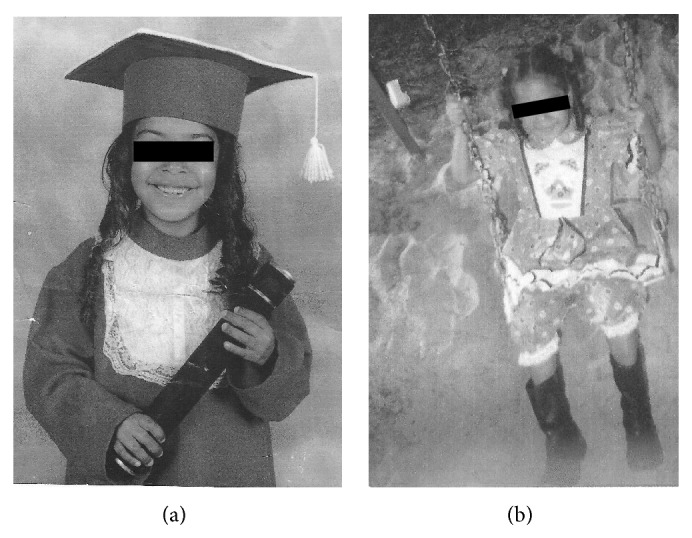
The healthy patient at 9 years of age.

An 11-year-old Caucasian female child with NPC disease was referred for dental treatment to the Human Resources Training Center at the Ribeirão Preto Dental School, University of São Paulo, Brazil. The center specializes in dental care for special needs patients. The patient presented with neonatal icterus; however, the patient was a full-term baby, underwent a normal delivery, and reached normal milestones until 9 years of age (Figures 1(a) and 1(b)). Her medical history revealed a first-degree consanguineous relationship between the parents.

**Figure 2:**
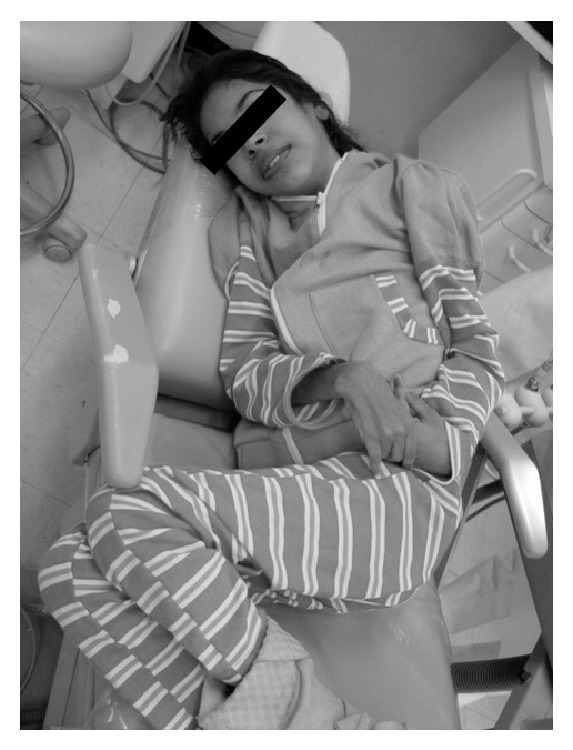
The patient's clinical appearance at 11 years of age included neuropsychomotor delay, dystonic posture of the hands and feet, and vertical supranuclear gaze palsy (VSGP).

NPC disease was first diagnosed at the age of 9 when the patient suffered a grand mal seizure, and other epileptic events gradually progressed. Subsequently, the patient presented with learning difficulties and progressive intellectual decline (Figure 2). The patient underwent videofluoroscopy, which showed lack of coordination between swallowing phases. After diagnostic confirmation, the patient was treated with Miglustat (N-butyldeoxynojirimycin, Zavesca, Actelion Pharmaceuticals Ltd., United Kingdom), Curcumin 95 (Curcumin, Demethoxycurcumin and Bisdemethoxycurcumin, Jarrow Formulas, Brazil), and Tegretol (Carbamazepina, Novartis, Brazil). The treatment resulted in a significant reduction in the progression of neurological symptoms.

**Figure 3:**
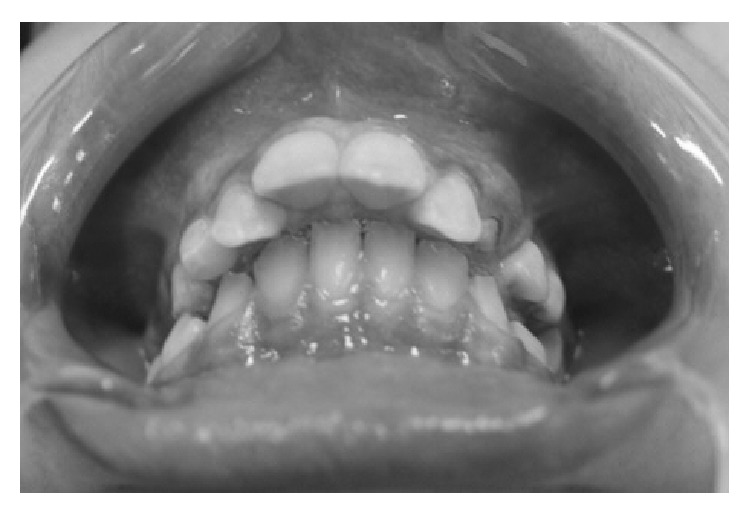
Oral findings revealing marked overjet and skeletal malocclusion angle class II, division I.

*Dental Findings. *The anamnesis and clinical exam revealed high caries risk and activity. The oral examination revealed the presence of poor oral hygiene, plaque biofilm, and supragingival calculus on the posterior teeth. Furthermore, the patient had halitosis, excessive salivation, enamel cracking of the maxillary central incisors, an incisal fracture in the right central incisor, marked overjet, skeletal malocclusion angle class II, division I (Figure 3), narrow palate, mobility in the upper primary molars, and caries in the first left and right permanent mandibular molars and first left premolar. 

**Figure 4:**
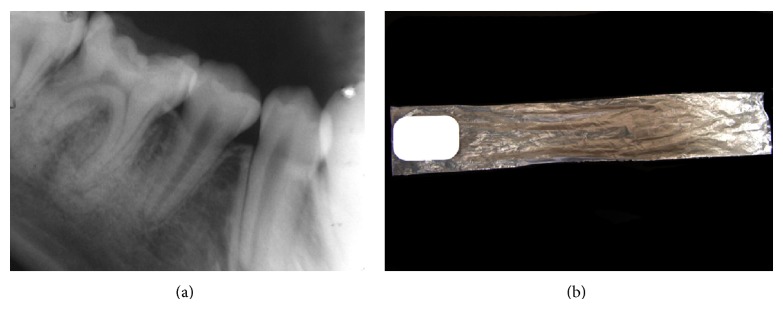
Periapical radiographic examination showing the presence of the first permanent molar with extensive caries and periodontal ligament thickening (a). An intraoral radiographic film enclosed in a plastic bag (4 × 23 cm) (b).

*Radiographic Examination. *Periapical radiographs showed extensive caries on the occlusal surface of the right permanent mandibular first molar accompanied by a slight thickening of the periodontal ligament (Figure 4(a)). The radiograph film was placed within a 4 × 23 cm plastic bag (Figure 4(b)) to control its position and to avoid the risk of swallowing or possible suffocation [12].

The present case report was conducted after receiving approval by the ethics committee at the School of Dentistry of Ribeirão Preto, USP (number 2010.1.1517.58.0).

## 3. Treatment

**Figure 5:**
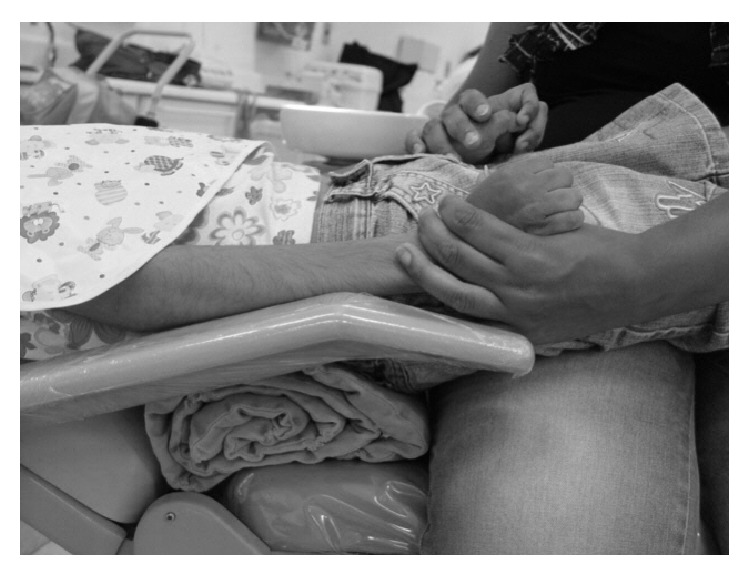
Containment of the child by the mother during the dental treatment.

The dental treatment was performed in an outpatient setting and in the presence of the mother, who helped in keeping the patient calm (Figure 5). Behavior management techniques were used, including tell-show-do, voice control, nonverbal communication, positive reinforcement, distraction [13], and desensitization [14]. A cork wrapped in a sterilized handkerchief was used to open the patient's mouth.

Preventive oral hygienic methods, including the correct technique for brushing the patient's teeth with the aid of an oral device to hold the mouth open, were explained to the mother. Dental prophylaxis was performed, followed by the application of topical fluoride.

Antisepsis of the oral cavity was performed by swabbing with 0.12% chlorhexidine before all of the dental procedures. The first and second primary molars of the maxillary right and left quadrants were extracted as a result of the advanced process of physiological root resorption and the risk of the patient swallowing the molars. The supragingival calculus was removed by quadrant using hand curettes and an ultrasonic apparatus (Profi II Ceramic, Dabi Atlante, Brazil) with high-power suction to avoid choking.

The dental treatment was performed following topical and infiltrative local anesthesia (Mepivacaine HCl 2% with 1 : 100,000 epinephrine) and rubber-dam isolation. The treatment of choice for the mandibular right first molar was pulpotomy. The pulp chamber was irrigated with sterile saline, and the coronal pulp was amputated at the level of the root canal entrances using sharp curettes. Hemostasis was obtained by copious irrigation of the pulp chamber with saline. For treatment, calcium hydroxide proanalysis (Calcium Hydroxide zur Analyse, Merck, Darmstadt, Germany) mixed with 0.5 mL of saline was applied and covered with a commercial calcium hydroxide cement layer (Dycal, Dentsply Indústria e Comércio Ltda) followed by amalgam restoration.

Resin restoration was the treatment of choice for the other teeth involved. After dental treatment was concluded, the patient returned to the dental clinic every 3 months for clinical evaluation and to receive prophylaxis with pumice, water, and a topical fluoride [13].

## 4. Follow-Up

**Figure 6:**
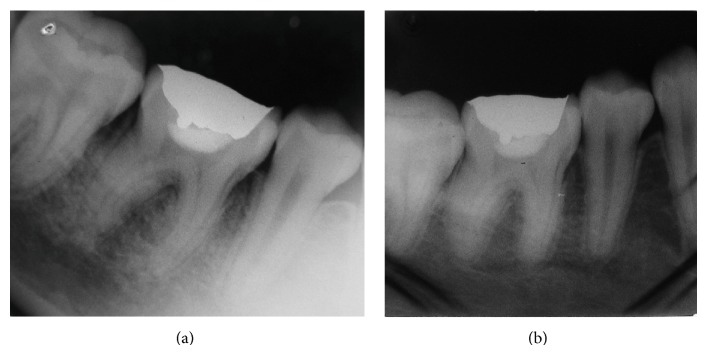
Two-year follow-up.

After 2 years, a new clinical and radiographic (Figure 6) examination was conducted on the tooth that received the pulpotomy to determine the absence of internal root resorption, bone rarefaction, sensitivity, pain, and swelling. The bacterial plaque biofilm was being adequately controlled by the caregiver.

## 5. Discussion

The current case report shows that caries risk and activity can be modified by a systemic disease. The patient was healthy and had a low caries risk until the age of 9, but when signs and symptoms of NPC appeared, the patient presented changes in diet and difficulties with toothbrushing. In addition, the caregiver required training to avoid the development of caries disease.

Caries appear in disabled children often because they do not chew. Diet and medications, such as sucrose syrup, are options that can improve chewing, but in the present case report, the patient used gavage for eating and to take medication.

No current reports have examined caries risk or oral findings that are associated with NPC. However, one report of Niemann-Pick disease type D found that gingival enlargement may be a possible manifestation of the disease [15]. This characteristic was not observed in our patient. The present patient presented dental caries that required preventive and restorative treatment. Children with special health care needs often present a high risk of caries activity [16]. In the present case, the parent was notified of this risk and was motivated to assist with daily oral hygiene. We also emphasized the importance of follow-up meetings every 2-3 months.

The entire dental treatment was performed on an outpatient basis, which prevented the child from undergoing general anesthesia. The mother actively participated in the dental treatment sessions, and the information she received during the meetings motivated her to participate more; subsequently, we observed an improvement in the oral hygiene that was performed by the mother for her child. In fact, with parent/caregiver assistance, most patients with physical and mental disabilities can be managed in the dental office [12]. In this case, creating a bond of trust with the patient yielded a progressive improvement of the patient's behavior during dental care. In addition to establishing a relationship with the patient, the use of communication techniques and positive reinforcement can help children to develop a positive attitude toward oral health and result in the appropriate use of dental hygiene. Dentists should consider the patient's cognitive development and the presence of communication deficits. The techniques of desensitization and specific methods of tell-show-do, voice control, nonverbal communication, positive reinforcement, and distraction are associated with positive outcomes [11, 14]. In our case, even though the patient had neurological development regression and cognitive and communication deficits, the techniques helped to improve the child's behavior.

Although panoramic and cephalometric radiography may complement the diagnosis of dental and craniofacial abnormalities that are associated with NPC, these techniques were not implemented in the present case, as the patient was unable to keep her head upright and steady during the radiography.

The periapical radiographic examination only included the tooth that was compromised by extensive dental caries. The examination was aided by behavior management techniques and protective stabilization. Cooperation from the person accompanying the patient was important for maintaining the film in the correct position in the oral cavity; this was facilitated by the use of a plastic device that decreased the child's risk of swallowing or choking on the radiographic film. Protective stabilization can be helpful in patients who do not respond to traditional behavior guidance techniques. With or without the aid of a restrictive device, protective stabilization can be performed by the dentist, staff, or a parent. The objectives of patient stabilization are to reduce or eliminate undesirable movement; to protect the patient, staff, dentist, and parent from injury; and to facilitate the delivery of quality dental treatment [11].

A pulpotomy with calcium hydroxide was used in the permanent mandibular right first molar, as studies have demonstrated the success of this technique [17–21]. For several years, calcium hydroxide has been the most widely accepted capping material for pulpotomies; the material possesses satisfactory biological properties and can induce the formation of a mineralized tissue barrier when placed in contact with the exposed pulp tissue [22–27]. In the present case, the tooth responded positively to a pulp sensitivity test (Endo Ice, Hygienic Corporation, Akron, Ohio), and there was no swelling of the support tissues. Furthermore, the patient's mother reported that the child showed no pain in that region. After the coronal opening, we observed a pulp tissue with macroscopic signs that were compatible with a vital tissue, which indicates a pulp with resistance to cutting and nonhemorrhagic bleeding. These characteristics are necessary for applying this type of treatment.

The earlier diagnosis of NPC and the early adoption of preventive measures are important to reduce and control the risk of caries activity.

## 6. Conclusion

In summary, risk factors associated with caries development are clearly altered by NPC diseases that affect healthy children. In a patient that presented with learning difficulties and a progressive intellectual decline, the present dental treatment was achieved without sedation by relying on techniques of behavioral management and desensitization. Preventive measures, education, caregiver training, and frequent consultations with the dentist are essential for patients with special health care needs.
